# Development of severe thrombocytopenia with TAFRO syndrome-like features in a patient with rheumatoid arthritis treated with a Janus kinase inhibitor: A case report

**DOI:** 10.1097/MD.0000000000022793

**Published:** 2020-10-16

**Authors:** Keiichiro Kadoba, Daisuke Waki, Keisuke Nishimura, Hiroki Mukoyama, Rintaro Saito, Hiroyuki Murabe, Toshihiko Yokota

**Affiliations:** Department of Endocrinology and Rheumatology, Kurashiki Central Hospital, Kurashiki, Okayama, Japan.

**Keywords:** case report, JAK inhibitor, rheumatoid arthritis, TAFRO syndrome

## Abstract

**Rationale:**

Thrombocytepenia, anasarca, fever, renal insufficiency, and organomegaly (TAFRO) syndrome is a novel disease entity characterized by a constellation of symptoms (thrombocytopenia, anasarca, fever, renal insufficiency, and organomegaly). Here, we describe the development of TAFRO syndrome-like features during the treatment of rheumatoid arthritis with a Janus kinase (JAK) inhibitor.

**Patient concerns:**

In this report, a 74-year-old woman treated with a JAK inhibitor (tofacitinib) for rheumatoid arthritis was admitted because of fever and thrombocytopenia.

**Diagnoses:**

On laboratory examination, marked thrombocytopenia and elevated creatinine and C-reactive protein levels were present. A computed tomography scan revealed lymphadenopathy, hepato-splenomegaly, and anasarca. A left axillary lymph node biopsy revealed Castleman's disease-like features. These clinical features satisfied the proposed diagnostic criteria for TAFRO syndrome. Since autoimmune disorders should be excluded when diagnosing TAFRO syndrome, it is not strictly correct to diagnose her as TAFRO syndrome. Therefore, we diagnosed her as rheumatoid arthritis complicated by TAFRO syndrome-like features.

**Interventions:**

The patient was treated with high-dose glucocorticoid, tacrolimus, eltrombopag, intravenous immunoglobulin, and rituximab.

**Outcomes:**

Her condition was refractory to the above-mentioned treatment, and she eventually died because of multi-organ failure 6 months after the first admission.

**Lessons:**

TAFRO syndrome-like features can develop during treatment with a JAK inhibitor for rheumatoid arthritis. Patients with autoimmune diseases complicated by TAFRO syndrome-like features can follow a fatal clinical course, and thus, an intensive combined treatment is warranted for such patients, especially in cases refractory to glucocorticoid.

## 1 Introduction

Thrombocytepenia, anasarca, fever, renal insufficiency, and organomegaly (TAFRO) syndrome is a systemic inflammatory disorder of undetermined etiology characterized by thrombocytopenia, anasarca, fever, renal insufficiency, and organomegaly. Diagnostic criteria for TAFRO syndrome have been recently proposed, and it requires all of the three major categories (anasarca, thrombocytopenia, and systemic inflammation) and at least two of four minor categories (Castleman's disease-like features on lymph node biopsy, reticulin myelofibrosis/increased number of megakaryocytes in bone marrow, mild organomegaly, and progressive renal insufficiency) to be present.^[1]^ Various other diseases, including autoimmune disorders, can fulfill the above-mentioned criteria, and careful exclusion is warranted during the application of such criteria in clinical practice.

Rheumatoid arthritis (RA) is a chronic, inflammatory joint disease, which can cause cartilage and bone damage as well as disability. Over half of RA patients test positive for autoantibodies against IgG (rheumatoid factor) or citrullinated peptides at diagnosis, which suggests an autoimmune etiology of the disease.^[2]^ Treatment of RA is usually initiated with conventional synthetic disease-modifying antirheumatic drugs (DMARDs) including methotrexate, but in refractory cases biological DMARDs or Janus kinase (JAK) inhibitors are effective treatment options. Tofacitinib is an oral JAK inhibitor for the treatment of RA. JAK is a tyrosine kinase that is essential for the signal cascade downstream of many pro-inflammatory cytokines. Tofacitinib modulates the immune response via down-regulation of several cytokines that are integral to lymphocyte development and function.^[3,4]^

Here, we present a case of a patient with RA treated with tofacitinib complicated by severe thrombocytopenia with TAFRO syndrome-like features.

## 2 Case presentation

A 74-year-old Japanese woman, who did not have a history of antecedent infection, was admitted to our hospital for fever and thrombocytopenia that had been present for one month. At the age of 69, she had been diagnosed with RA based on a positive anti-cyclic citrullinated peptide antibody and rheumatoid factor test results, and multiple small joint synovitides and erosions detected by ultrasound. She was treated with tofacitinib and had been in remission for the past several months. She had a history of allergy to tocilizumab.

**Figure 1:**
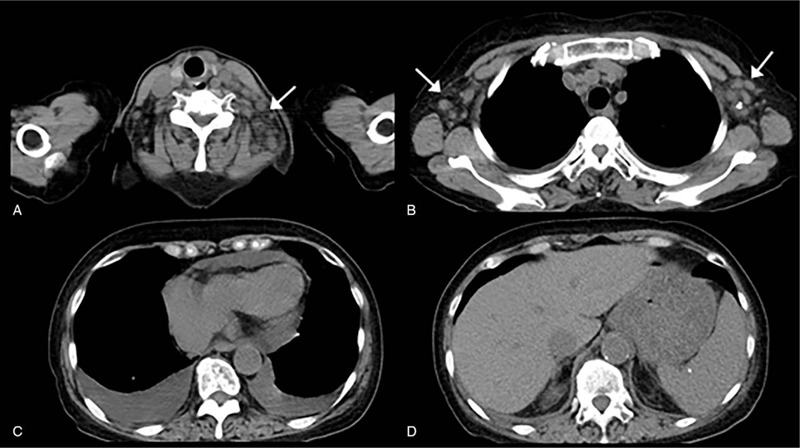
Computed tomography scan reveals cervical lymphadenopathy (A, arrow), bilateral axillary lymphadenopathy (B, arrow), bilateral pleural effusion, pericardial effusion (C), and hepato-splenomegaly (D).

**Figure 2:**
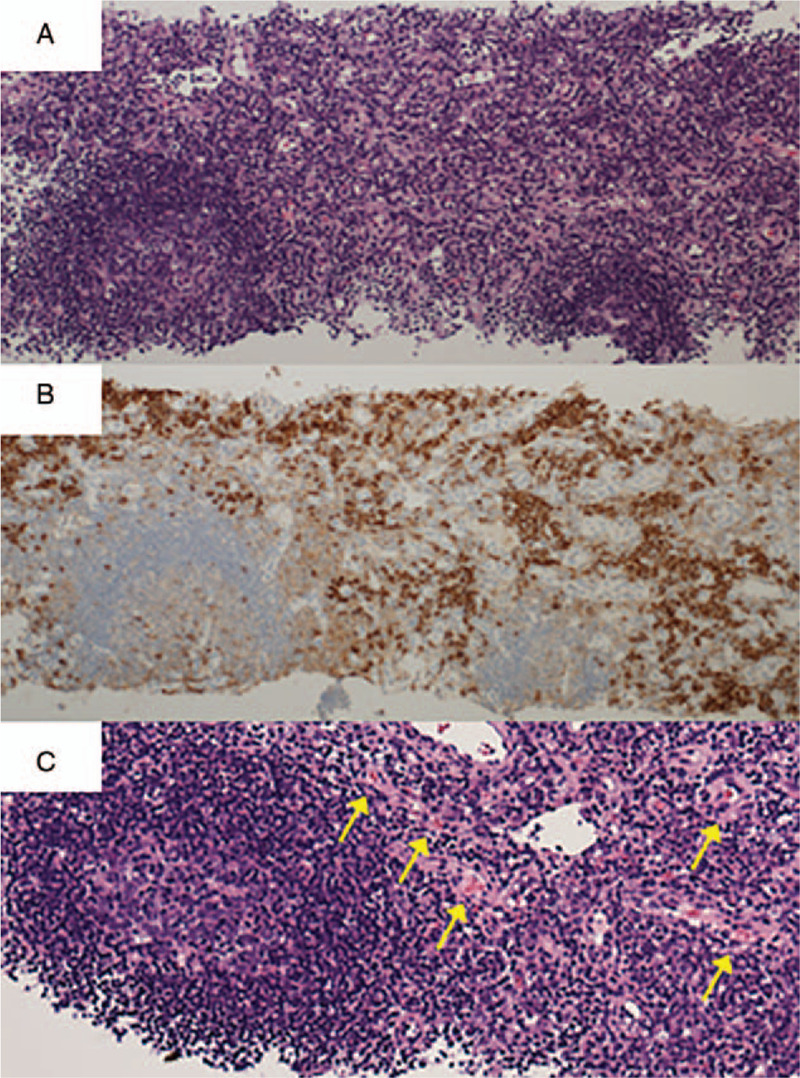
Histological findings of the axillary lymph node. (A) Atrophic germinal centers and the expansion of the interfollicular zone. Hematoxylin and eosin staining (100 × magnification). (B) Scattered CD38-positive plasma cells in the interfollicular area. Immunohistochemical staining using an anti-CD38 antibody (100 × magnification). (C) Proliferation of small vessels (yellow arrow) are observed in the interfollicular area. Hematoxylin and eosin staining (200 × magnification).

**Figure 3:**
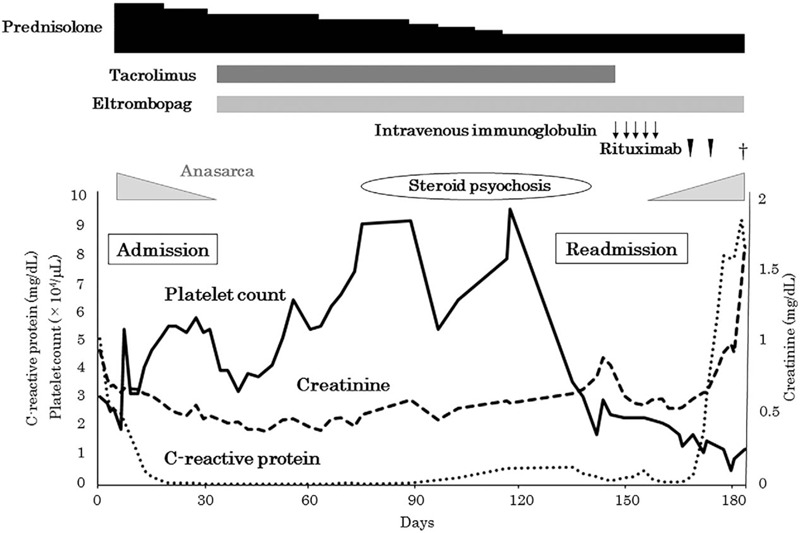
Clinical course of the case.

On admission, her vital signs were unremarkable. Physical examination revealed swollen lymph nodes in the neck and pitting edema in her legs. Laboratory examination findings were as follows: hemoglobin 8.3 g/dL, platelet count 34 × 10^3^/μL, serum creatinine 0.92 mg/dL, and C-reactive protein (CRP) 4.98 mg/dL. Her serum anti-nuclear antibody test was positive (80 folds, speckled pattern; 640 folds, centromere pattern), and her anti-RNP, anti-centromere, and anti-mitochondrial M2 antibody tests were also positive. Her urinalysis result was unremarkable. Screening for infection, which included cytomegalovirus and Epstein Barr virus, was also unremarkable. A computed tomography (CT) scan revealed cervical lymphadenopathy, bilateral axillary lymphadenopathy, bilateral pleural effusion, pericardial effusion, small ascites, and hepato-splenomegaly (Fig. 1). A left axillary lymph node biopsy revealed proliferation of small vessels and scattered CD38-positive plasma cells in the interfollicular area and atrophic germinal centers along with small vessels entering the germinal centers, without any findings suggestive of malignant lymphoma (Fig. 2). These findings were compatible with the histology of mixed-type Castleman's disease. Bone marrow biopsy did not show specific findings. Her clinical presentation (thrombocytopenia, anasarca, fever, elevated CRP and creatinine level, hepato-splenomegaly, and lymphadenopathy) and the histological findings of the axillary lymph node were compatible with TAFRO syndrome. Additional laboratory test findings showed serum interleukin-6 levels of 12.0 pg/mL (normal < 4.0 pg/mL), and vascular endothelial growth factor levels of 67.0 pg/mL (normal < 38.3 pg/mL). Oral prednisolone (50 mg/day) was initiated, and anasarca and systemic inflammation rapidly resolved. From the patient's perspective, her fatigue was dramatically improved. Platelet count also initially recovered, but decreased again upon tapering prednisolone. Tacrolimus and eltrombopag were added to the treatment. Platelet levels gradually increased thereafter and she was discharged (Fig. 3).

On her first outpatient visit after discharge, the patient had developed steroid psychosis and thus prednisolone was rapidly tapered. After 2 months, her platelet count decreased to 49 × 10^3^/μL and she was readmitted for further examination and treatment.

On the second admission, laboratory tests showed thrombocytopenia (24 × 10^3^/μL), microcytic anemia (hemoglobin, 9.6 g/dL), and slightly elevated CRP level (0.61 mg/dL). A CT scan revealed only mild cervical lymphadenopathy and splenomegaly, whereas pleural effusion, pericardial effusion, and ascites were absent. Reinduction with high-dose prednisolone was avoided because of suspected steroid psychosis, and intravenous immunoglobulin was administered for thrombocytopenia, with little effect.

One month after the second admission, her platelet count decreased to 17 × 10^3^/μL. A CT scan revealed bilateral pleural effusion, pericardial effusion, and exacerbation of systemic lymphadenopathy and hepato-splenomegaly. Rituximab was initiated at a dose of 375 mg/m^2^ weekly, with little effect on her thrombocytopenia, anasarca, and organomegaly. Her general condition gradually deteriorated, and she died due to multi-organ failure compatible with TAFRO syndrome, two months after the second admission.

Patient consent was obtained from family members for this case report.

## 3 Discussion

This is the first case of RA complicated by thrombocytopenia with TAFRO-like features. To date, there have been only 6 reported cases of autoimmune diseases complicated by TAFRO-like features (3 cases of Sjögren's syndrome,^[5–7]^ 1 case of Sjögren's syndrome and systemic sclerosis,^[8]^ 1 case of systemic lupus erythematosus,^[9]^ and 1 case of ulcerative colitis^[10]^), but no cases of concomitant RA and TAFRO-like features have been reported.

There have been two reported cases of RA complicated by multicentric Castleman's disease (MCD).^[11,12]^ TAFRO syndrome may be considered a subtype of MCD due to the similarity of histological findings, but recently TAFRO syndrome has been proposed as a distinct disease entity with some features overlapping with MCD. Distinguishing features of TAFRO syndrome include thrombocytopenia, normal γ-globulin levels, elevation of serum alkaline phosphatase, anasarca, and coagulation abnormality. In contrast to MCD, which usually has a chronic clinical course, TAFRO syndrome develops sub-acutely, progresses rapidly, and can often prove fatal.^[13]^ Moreover, TAFRO syndrome was recently reported to have unique histological features (atrophic germinal center along with high endothelial venules in the germinal center and interfollicular zone, and small numbers of plasma cells in the interfollicular zone), which can be helpful in making a definitive diagnosis.^[14]^

In the present case, physical examination, laboratory findings, image findings, and histological findings are compatible with TAFRO syndrome and satisfy 3 major categories and three minor categories of the proposed diagnostic criteria for TAFRO syndrome,^[1]^ although reticulin myelofibrosis was not proven on bone marrow biopsy. In addition, in our case, the findings of left axillary lymph node biopsy are compatible with the above-mentioned histological features of TAFRO syndrome, and thus it is reasonable to describe our case as thrombocytopenia with “TAFRO-like features.”

Her condition may satisfy the 2012 Systemic Lupus International Collaborating Clinics classification criteria (synovitis, serositis, thrombocytopenia and antinuclear antibody).^[15]^ However, the constellation of symptoms typical of TAFRO syndrome, the absence of anti-double-stranded DNA and anti-Smith antibodies, normal levels of serum complement, and especially the characteristic histological findings of the lymph node biopsy suffice to describe our case as thrombocytopenia with “TAFRO-like features.” Malignant lymphoma and immune thrombocytopenic purpura can also complicate RA, but the clinical course of our case is hardly explicable by these disorders.

Interestingly, our patient developed TAFRO-like features during treatment with tofacitinib. No case of TAFRO syndrome or Castleman's disease following tofacitinib treatment has been reported. Development of TAFRO-like features during potent immunosuppression with tofacitinib suggests that TAFRO syndrome can partly be driven by a non-autoimmune mechanism. One proposed etiology of TAFRO syndrome is viral infection. A fraction of multicentric Castleman's disease cases, which shares some clinical features with TAFRO syndrome, are associated with human herpesvirus-8.^[16]^ Moreover, Simons et al reported a case of biopsy-proven Epstein-Barr virus-positive TAFRO syndrome successfully treated with a three-drug regimen, including tocilizumab, etoposide, and rituximab.^[17]^ Considering the high incidence of varicella zoster virus reactivation during tofacitinib treatment,^[3]^ latent virus reactivation might have contributed to the development of TAFRO-like syndrome in our case.

Considering the complete clinical picture, we decided to treat our patient in accordance with the treatment strategy for TAFRO syndrome. High-dose glucocorticoid is the first-line treatment for TAFRO syndrome^[1]^. In refractory cases, the addition of immunosuppressive agents, such as calcineurin inhibitors (i.e., cyclosporin A or tacrolimus), tocilizumab, and rituximab, has been used to control symptoms.^[1,18]^ Thrombopoietin receptor agonists have also been used in patients with persistent thrombocytopenia.^[19,20]^ Although we tried all the above options, except for tocilizumab, due to her allergy to it, our patient followed a fatal clinical course. Fujimoto et al reported that, despite intensive immunosuppressive treatment, one-third of patients with TAFRO syndrome die within two years, especially during the first few months.^[13]^ Further accumulation of cases is required in order to achieve better survival rates of this potentially fatal disease.

In conclusion, we reported a case of RA complicated by refractory thrombocytopenia with TAFRO-like features during treatment with tofacitinib. We should keep in mind that patient with autoimmune diseases, such as RA, can develop complications of TAFRO-like disorders, because prompt and intensive immunosuppressive treatment should be considered in such cases.

## Acknowledgments

We would like to thank Editage (http://www.editage.com) for English language editing.

## Author contributions

**Validation**: Hiroki Mukoyama, Rintaro Saito, Hiroyuki Murabe, Toshihiko Yokota.

**Writing – original draft**: Keiichiro Kadoba.

**Writing – review & editing**: Keiichiro Kadoba, Daisuke Waki, Keisuke Nishimura.
